# Malignant phosphaturic mesenchymal tumor of the pelvis: A report of two cases

**DOI:** 10.3892/ol.2014.2081

**Published:** 2014-04-22

**Authors:** TOKIMITSU MORIMOTO, SATOSHI TAKENAKA, NOBUYUKI HASHIMOTO, NOBUHITO ARAKI, AKIRA MYOUI, HIDEKI YOSHIKAWA

**Affiliations:** 1 Department of Orthopedic Surgery, Osaka University Graduate School of Medicine, Osaka 565-0871, Japan; 2 Department of Orthopedic Surgery, Bell Land General Hospital, Osaka 599-8247, Japan; 3 Osaka Medical Center for Cancer and Cardiovascular Diseases, Osaka 537-8511, Japan; 4 Medical Center for Translational Research, Osaka University Hospital, Osaka 565-0871, Japan

**Keywords:** phosphaturic mesenchymal tumor, osteomalacia, transformation, malignant, fibroblast growth factor 23

## Abstract

Tumor-induced osteomalacia (TIO) is a rare acquired form of hypophosphatemia commonly associated with phosphaturic mesenchymal tumors (PMTs) located in the bone or soft tissue. Resection of the tumor can cure osteomalacia. Fibroblast growth factor 23 has been identified as a major pathophysiological factor responsible for phosphaturia. The majority of PMTs are benign, and malignant PMTs are uncommon. Even in rare cases, the malignant transformation of PMTs is extremely uncommon. The current study presents two cases in which the patients succumbed to malignant PMTs that developed in the pelvis. The first patient was a 35-year-old female with a malignant PMT occurring as a synchronous double cancer associated with papillary thyroid carcinoma. Diagnosis was difficult, as the multiple uptake on positron emission tomography with 18F-fluorodeoxyglucose presented as pseudofractures mimicking the metastases of thyroid carcinoma. The patient succumbed to rapidly progressive lung metastases. The second patient presented with a pelvic tumor that had developed over 26 years. The patient was diagnosed with a benign PMT by open biopsy and a complete resection was performed. However, two years later, the tumor recurred and lung metastases were observed. The patient ultimately succumbed to respiratory failure due to relapsing lung metastases and disseminated intravascular coagulation. These two cases demonstrate the potential lethality of malignant PMTs and the malignant transformation of benign PMTs. Therefore, TIO patients must be followed up even if diagnosed with a benign tumor. Although TIO is an extremely rare disease, the possibility of malignant PMTs must be recognized.

## Introduction

Tumor-induced osteomalacia (TIO) is an acquired type of hypophosphatemia that is frequently associated with mesenchymal tumors (1). In 1947, McCance *et al* described the first case of TIO, and ~300 cases have been reported in the literature (1,2). TIO is characterized clinically by fractures, bone pain, phosphaturia, hypophosphatemia, low serum 1.25(OH)_2_D concentrations and high serum alkaline phosphatase (ALP) concentrations (1,3,4). Fibroblast growth factor 23 (FGF-23) has been identified as a major pathophysiological factor responsible for phosphaturia (1,5–7). Surgical resection of the tumor results in the dramatic improvement of symptoms in the majority of cases (1,4). In 1987, Weidner *et al* (8) described the pathological features of a series of 17 mesenchymal tumors classified as a single entity that caused TIO, and labeled them as phosphaturic mesenchymal tumors (PMTs). However, the majority of clinicians and pathologists are not aware of the existence of this type of tumor, and it is often misdiagnosed as another type of tumor. The majority of PMTs are benign, and malignant PMTs resulting in mortality are extremely rare. The current study presents the cases of two patients who succumbed to malignant PMTs of the pelvis. Patients provided written informed consent.

## Case report

### Case one

**Figure 1:**
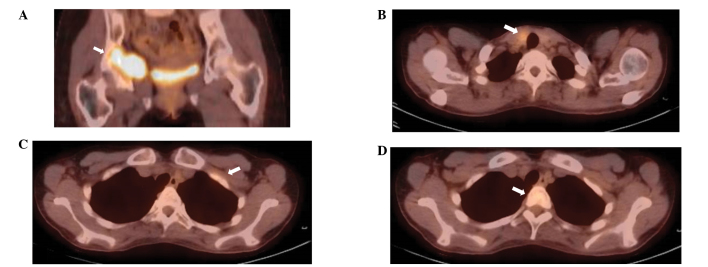
Case one: Positron emission tomography with ^18^F-fluorodeoxyglucose (FDG-PET) showing abnormal uptake in the (A) right acetabulum, (B) right lobe of the thyroid gland, (C) left first rib and (D) T2 vertebral body, which were considered to be multiple bone metastases from the thyroid cancer.

**Figure 2:**
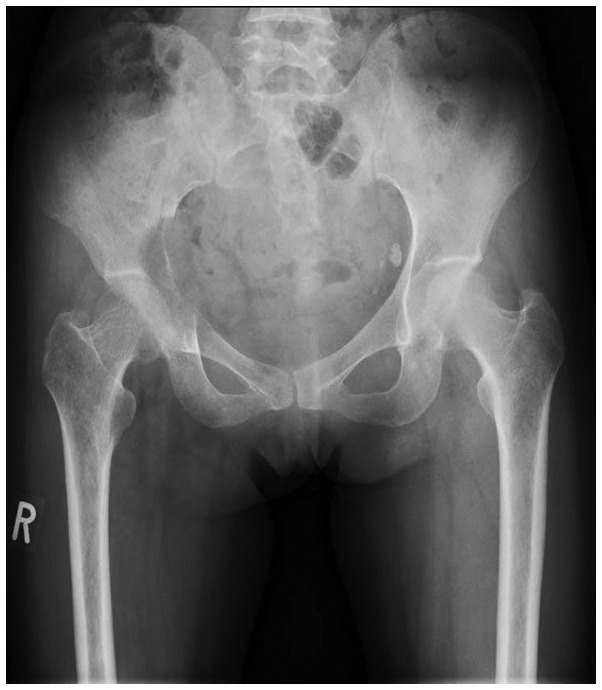
Case one: Plain radiograph showing an osteolytic lesion in the right acetabulum.

**Figure 3:**
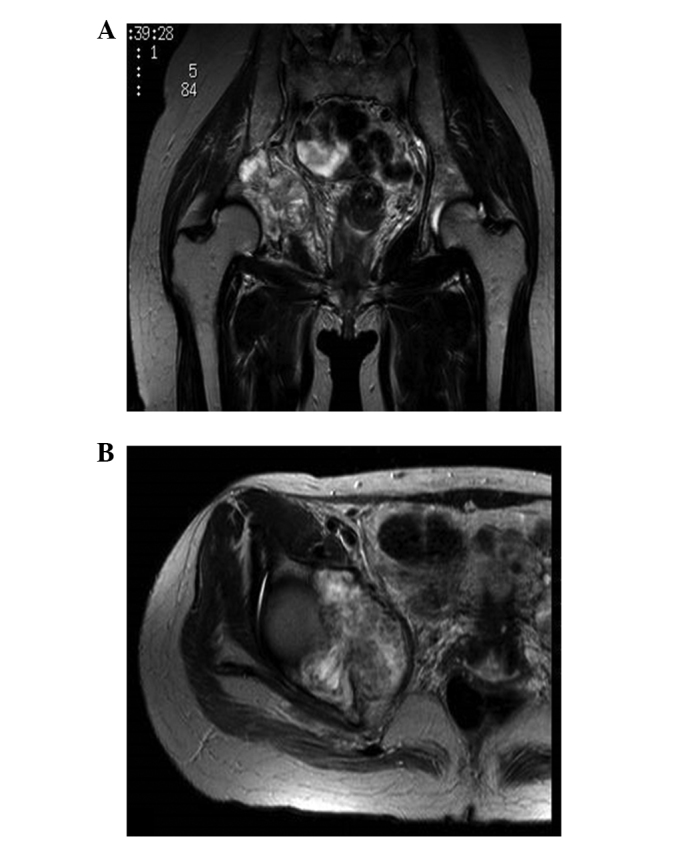
Case one: (A) Coronal and (B) axial T2-weighted magnetic resonance imaging (MRI) showing a mass with inhomogeneous intensity located in the right acetabulum.

**Figure 4:**
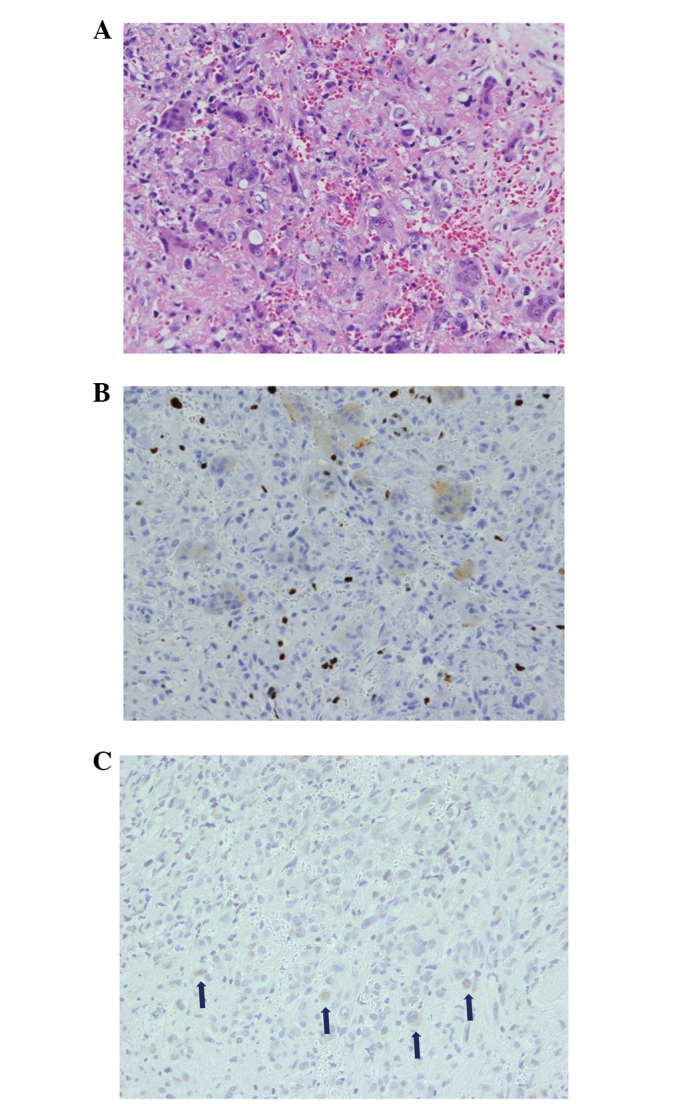
Case one: Open biopsy of the acetabular lesion. (A) Histological examination revealed spindle and round cells, with the multinucleated giant cells in a collagenous matrix with capillaries (hematoxylin-eosin stain; magnification, ×400). In the immunological studies (B) the Ki-67 index was 20% and (C) the tumor cells were fibroblast growth factor 23-positive (indicated by the arrows; immunohistochemical stain; magnification, ×200). Based on these observations, the tumor was diagnosed as a malignant phosphaturic mesenchymal tumor (PMT).

In March 2008, a 35-year-old female was referred to the Osaka University Hospital (Osaka, Japan) with lower back pain. Laboratory analysis revealed high ALP levels (1,080 mg/ml), with severe hypophosphatemia (1.4 mg/ml), high thyroglobulin (Tg) levels (65.2 mg/ml), normal calcium levels (8.7 mg/dl) and normal parathyroid hormone levels (58.7 mg/ml). Upon physical examination, a mass was identified in the right neck, and a needle biopsy of the mass revealed a papillary thyroid carcinoma. On positron emission tomography with ^18^F-fluorodeoxyglucose (FDG-PET), abnormal uptake was observed in the right pelvis (maximum standardized uptake value of 7.0), left first rib, T2 vertebra and right lobe of the thyroid gland (Fig. 1). X-ray examination revealed an osteolytic lesion in the right pelvis (Fig. 2), and T2-weighted magnetic resonance imaging (MRI) revealed a mass with inhomogeneous intensity in the right acetabulum (Fig. 3). The diagnosis was of multiple bone metastases from papillary thyroid carcinoma. Radiation therapy to the right pelvis (40 Gy/20 fractions) followed a total thyroidectomy. However, the serum Tg levels normalized completely following thyroidectomy, despite the presence of multiple lesions considered to be metastases. Bone scintigraphy revealed multiple linear hot spots over the ribs, as frequently observed with pseudofractures in osteomalacia. Subsequently, an open biopsy of the pelvic lesion was performed. Histopathology showed spindle and round cells, with multinucleated giant cells in a collagenous matrix with capillaries (Fig. 4A). Immunological studies revealed FGF-23-positive tumor cells and a Ki-67 index of 20% (Fig. 4B and C). In addition, the serum FGF-23 levels were elevated to 121 pg/ml (reference range, 10–50 pg/ml). Based on these findings, the tumor was diagnosed as a malignant PMT. The patient rejected wide resection of the pelvic tumor with reconstructive total hip arthroplasty. Subsequently, transcatheter arterial embolization (TAE) of the feeding artery of the pelvic tumor was performed, and the patient was administered disodium phosphate (2 g/day) and vitamin D (alphacalcidol; 2 μl/day) with monitoring serum phosphate and 1.25(OH_2_)D co- ncentrations. The tumor decreased in size after TAE had been performed twice. The serum phosphate and ALP levels were gradually normalized, and the multiple uptake on FDG-PET also disappeared, with the exception of the pelvic lesion, which indicated that this uptake was due to pseudofractures as opposed to malignancies. However, regrowth of the pelvic tumor and multiple metastases in the lung and bones were observed 32 months after the second TAE. In addition, leukocytosis (24,950 cells/ml), without C-reactive protein elevation, and a high level of granulocyte colony-stimulating factor (G-CSF; 713 pg/ml) were observed. It is possible that the tumor had been transformed from an FGF-23-producing tumor to a G-CSF-producing tumor. Chemotherapy consisting of combined Adriamycin (55 mg/m^2^) and ifosfamide (8 g/m^2^), and combined gemcitabine (900 mg/m^2^) and docetaxel (75 mg/m^2^), was administered, however no effect was observed and the patient succumbed to rapidly progressive lung metastases.

### Case two

**Figure 5:**
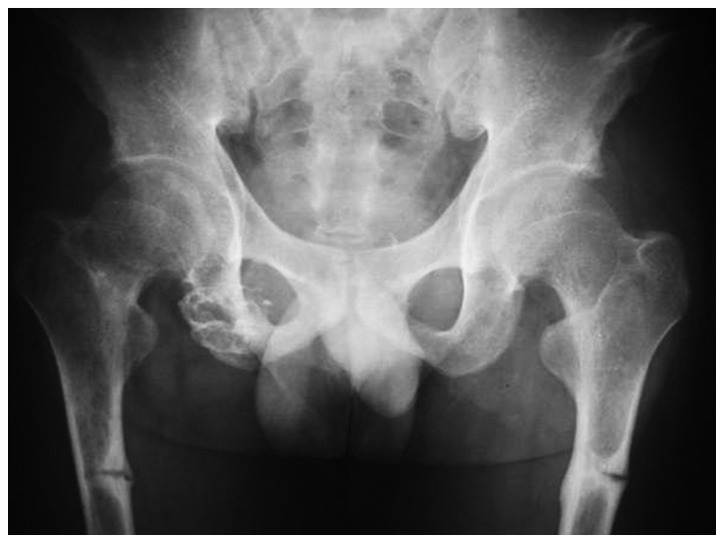
Case two: Plain radiograph showing a bony destructive lesion, with calcification in the right ischium and ununited fractures in the shafts of the bilateral femora (Looser’s zones).

**Figure 6:**
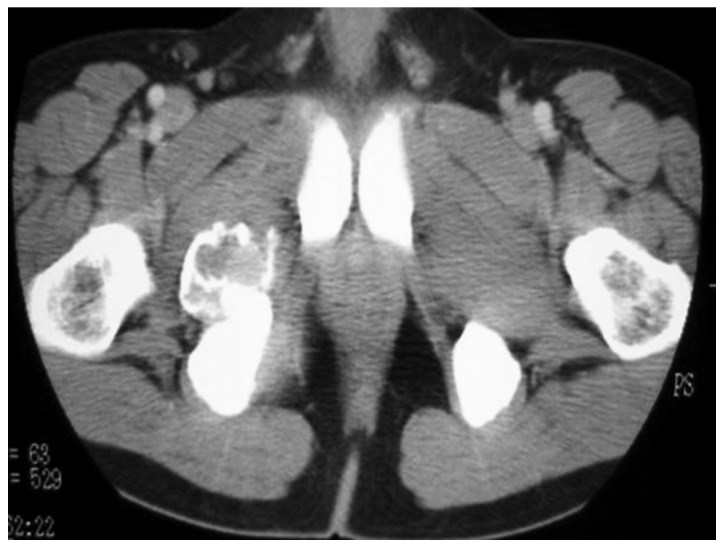
Case two: Contrast-enhanced computed tomography showing a homogeneously-enhanced mass in the pelvis.

**Figure 7:**
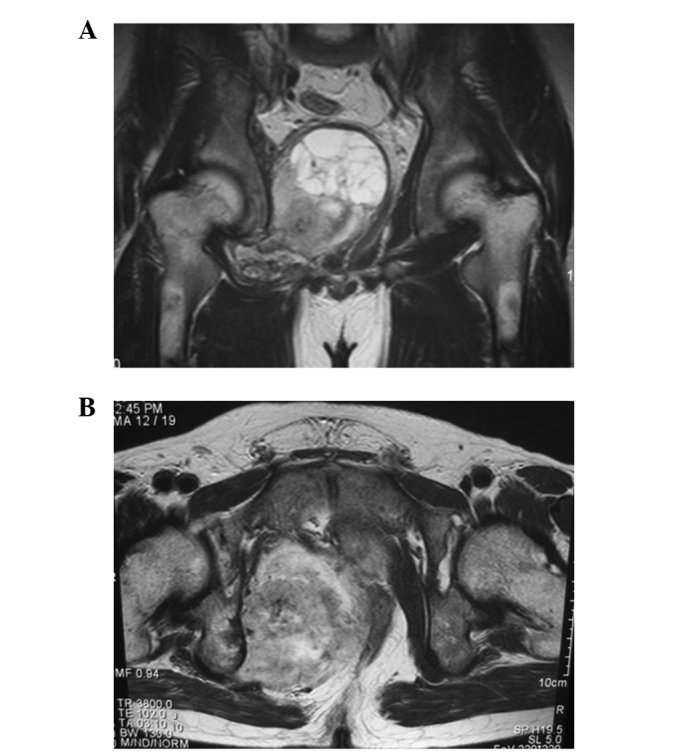
Case two: (A) Coronal and (B) axial T1-weighted gadolinium-enhanced magnetic resonance imaging (MRI) revealing a heterogeneous intensity mass, with partial cystic change, compressing the rectum and bladder.

In 1982, a 10-year-old male was originally diagnosed with hypophosphatemic osteomalacia of unknown cause. At 25 years old, an intrapelvic tumor was incidentally found, however, the patient did not undergo a detailed examination due to a lack of enlargement in the subsequent two years. In December 2003, at 31 years old, the patient visited the Osaka Koseinen-kin Hospital (Osaka, Japan) due to worsening bilateral thigh pain and gait disturbance. A TIO was suspected and as a result, the patient was referred to the Osaka Medical Center for Cancer and Cardiovascular Diseases (Osaka, Japan). The laboratory analysis revealed severe hypophosphatemia (1.3 mg/ml), accompanied by high ALP (1,648 mg/ml) and FGF-23 (3,319 ng/ml) levels. X-ray examination revealed a bony destructive lesion, with calcification in the right ischium and ununited fractures in the shaft of bilateral femora, indicating Looser’s zones (Fig. 5). In addition, contrast-enhanced computed tomography (CT) revealed an inhomogeneously enhanced mass in the pelvis (Fig. 6). The tumor had increased in size since its discovery when the patient was 25 years old, and MRI revealed a heterogeneous intensity mass with partial cystic change compressing the rectum and bladder (Fig. 7). A bone scan showed multiple abnormal accumulations, indicating multiple pseudofractures. An open biopsy was subsequently performed. The histopathology of the pelvic lesion revealed spindle cells in a collagenous and cartilaginous matrix, with vasculature, but without cytological atypia or mitosis. The diagnosis was of a benign PMT of the mixed connective tissue (MCT) type. The patient underwent two courses of TAE of the feeding artery of the pelvic tumor, with only limited response, and therefore subsequently underwent tumor excision. The tumor was completely resected with tumor-free margins, and the serum phosphorus levels were normalized within 10 days after surgery. Furthermore, on radiographs, the bilateral femoral pseudofractures were shown to gradually heal. However, two years after the surgery, follow-up blood tests revealed elevated serum FGF-23 (230 pg/ml) levels, and CT and MRI showed local recurrence in the pelvis. In addition, FDG-PET CT showed local recurrence in the pelvis, multiple coin lesions in the lung and a subcutaneous mass in the left elbow with FDG uptake. Metastases of the PMT were diagnosed clinically and the patient underwent chemotherapy, including two courses of gemcitabine (1,000 mg/m^2^) and docetaxel (120 mg/m^2^), with no effect. The patient then developed metastases in the bilateral lungs, bones and liver. Upon pathological analysis, the diagnosis was of metastases from the PMT with malignant transformation. The patient underwent liver metastasis resection, followed by two courses of Adriamycin (59 mg/m^2^) and ifosfamide (7 g/m^2^), and two courses of Adriamycin (71 mg/m^2^) and cisplatin (76 mg/m^2^). A complete response of the lung lesion was achieved briefly, whereas the intrapelvic tumor continued to grow despite radiation therapy and TAE. In addition, the skin of the buttocks became thinner, ultimately forming a malignant ulcer. The patient succumbed to respiratory failure due to relapsing lung metastases and disseminated intravascular coagulation.

## Discussion

The first case presented in the current study was difficult to diagnose, as the malignant pelvic PMT presented as a synchronous double cancer with thyroid carcinoma. TAE was selected, as the patient was unable to accept the functional impairment that would be a result of surgery. Although TAE safely achieved a temporary cytoreductive effect without massive release of FGF-23, 32 months after the second course of TAE, regrowth of the pelvic tumor and multiple metastases emerged. The patient succumbed to the rapid progression of the lung metastases.

The second case remained undiagnosed for a long period, as TIO was not commonly recognized at the time. Therefore, when the patient was eventually diagnosed with TIO, the tumor was too large to perform a wide resection with a curative margin. Upon analysis of a biopsy, the initial diagnosis was of a benign PMT-MCT, however, two years after surgery, multiple metastases appeared and resection of the liver metastasis revealed malignant PMT-MCT.

Ogose *et al* (9) reported local recurrence and malignant transformation from benign PMT in the course of long-term follow-up. Therefore, TIO patients must be followed up even if diagnosed with a benign tumor. In certain cases of TIO, it is difficult and time-consuming to detect the tumor inducing the osteomalacia (9–15). However, it is important to identify the tumor, as it may be malignant or change from benign to malignant. Previous studies have demonstrated the usefulness of FDG-PET CT in detecting the tumor inducing the osteomalacia (16–20). However, FDG accumulation may also occur in a pseudofracture, as observed in case one of the current study. Therefore, it is important to distinguish multiple metastases and pseudofractures from TIO.

With regard to chemotherapy, few studies have investigated the chemotherapeutic treatment of malignant PMT (21,22). Seijas *et al* (21) reported second distant metastases, however, the use of six cycles of Adriamycin stabilized the metastasis sites for two years. Sidell *et al* (22) reported only a limited response to chemotherapy consisting of doxorubicin, docetaxel and gemcitabine, however, significant tumor destruction was observed histologically. In case two of the current study, a complete response of the metastatic lung lesion was temporarily achieved using an Adriamycin-based regimen. Therefore, Adriamycin may exhibit tumor suppressive activity, however, additional evaluations are required.
